# Impact of Mobility Loss on Independence and Psychosocial Wellbeing in People With Parkinson’s Disease and Carers: A Meta-Ethnography

**DOI:** 10.1177/08919887251414664

**Published:** 2026-01-07

**Authors:** Megan Hanrahan, Cameron Wilson, Lynn Rochester, Alison Keogh, Ríona Mc Ardle, Katie Brittain

**Affiliations:** 1 Population Health Sciences Institute, 5994 Newcastle University, Newcastle Upon Tyne, England; 2 School of Clinical Medicine, Department of Public Health and Primary Care, 2152 University of Cambridge, Cambridge, England; 3 Translational and Clinical Research Institute, 5994 Newcastle University, Newcastle Upon Tyne, England; 4 Clinical Medicine, School of Medicine, 8809 Trinity College Dublin, Dublin, Ireland

**Keywords:** Parkinson’s disease, mental health, caregiver burden

## Abstract

**Background:**

Mobility impairments in Parkinson’s disease (PD) significantly impact individuals’ physical health, independence, and psychosocial wellbeing. In this review, mobility loss is used to refer to altered or impaired mobility (eg, reduced gait, balance difficulties, freezing of gait), rather than a complete loss of mobility. These changes affect daily functioning and quality of life. Carers also experience significant burden as they manage mobility issues. Understanding the implications of mobility loss in PD is essential for improving support for both people with PD and carers. This review explores impact of mobility loss on perceived independence and psychosocial wellbeing in people with PD and carers, providing deeper insight into the broader emotional and social consequences of the condition.

**Methods:**

A systematic search of six health databases (Medline, Embase, PsycInfo, CINAHL, Scopus, Web of Science) was conducted using key terms related to mobility loss, independence, and psychosocial wellbeing in PD. Studies were included if they employed qualitative methods to explore experiences of mobility from people with PD and/or carers. The methodological quality of studies was assessed using the NICE qualitative checklist, and reporting adhered to PRISMA guidelines. A meta-ethnographic approach was used to extract key themes and construct a comprehensive understanding of the findings.

**Results:**

Five key themes emerged: the struggle for independence and associated self-esteem, navigating personal relationships, perceived stigmatisation and social isolation, resilience and adaptation, and impact of mobility on carer wellbeing.

**Discussion:**

Findings highlight the need for comprehensive support systems that address the challenges of mobility loss in PD. Wearable technology presents a promising solution for personalised interventions. Future research should explore diverse populations of people with PD and include formal carers to develop a more holistic perspective of mobility-related challenges in PD caregiving.

## Introduction

Functional abilities may decline during ageing, leading to some degree of functional dependency.^1^ Physical mobility, defined as the ability to move independently from one place to another through functional movements like walking, is a central function in maintaining overall wellbeing.^2-6^ Mobility is associated not only with physical health but also mental, emotional, and psychological wellbeing, and is pertinent to one’s identity and sense of self.^6^ While mobility limitations are often identified in older adults, certain clinical populations with mobility-related impairments experience a relatively early and accelerated decline.^7,8^ For instance, Parkinson’s disease (PD) is a neurodegenerative condition characterised by progressive motor dysfunctions which impair mobility and cause rigidity in one’s movement, significantly impacting quality of life.^9-11^ These mobility limitations have significant social and emotional consequences for people with PD, such as reduced quality of life, increased depressive symptoms, and restricted social participation.^12-14^

The impact of mobility loss in PD extends beyond the individual, profoundly affecting carers as well. PD is a complex condition that encompasses multiple limitations, including cognitive impairments, autonomic dysfunction, and mobility loss.^15,16^ Caring for someone with PD can place significant burden on carers, often resulting in psychological distress.^17,18^ Research shows that 40-50% of PD carers report symptoms of anxiety and depression,^17^ and that the prevalence of depressive disorders among carers increased from 11.6% to 17.8% over a 12-month period^18^; these statistics demonstrate the considerable psychological strain associated with caregiving. The burden is particularly pronounced when caring for people with more severe mobility impairments, as these cases often require increased physical assistance and heighten emotional stress due to higher dependency,^19-21^ further demonstrating that mobility loss plays a central role in the caregiving experience. Carer burden not only diminishes the carers’ quality of life but can also lead to reduced care capacity or increase the likelihood of an earlier transition to formal care for the person with PD. Such transitions not only pose wider economic and public health challenges,^22-24^ but also impact the person’s independence and wellbeing, and can place added emotional strain on families. While some syntheses have addressed carer burden in PD, they often consider the condition in its entirety. Several reviews have already explored important aspects of the lived experience of PD, including stigma,^25^ psychosocial impact,^26^ relationships,^27^ identity and quality of life.^28^ These reviews demonstrate the breadth of challenges faced by people with PD and carers; however, mobility loss is often treated as one theme among many rather than the central analytical focus. Despite existing research, there is a need for a more targeted analysis that looks at the experience of mobility loss as a defining feature of the PD experience. Understanding mobility-related challenges could not only highlight the specific burdens faced by carers but also ensure we monitor meaningful aspects of mobility for both people with PD and carers. This insight is essential for guiding timely and targeted interventions that support mobility, potentially alleviating carer strain and improving quality of life for both groups.

The value of exploring lived experience is being increasingly acknowledged in health and care research.^29^ It illuminates both the perspectives and experiences of affected communities and is perhaps one of the most useful forms of research in this context, as those impacted by PD understand most about the complications of dealing with it.^30-32^ Therefore, investigations into the lived experience of PD have increased, demonstrated by two recent meta-ethnographies.^33,34^ While these reviews offer valuable insights and informs us of factors that contribute to the PD experience, their primary focus was on the broader impact on quality of life. Despite touching on psychological distress and social challenges, they did not fully identify how mobility loss specifically relates to these wider challenges. Interestingly, none of the existing reviews define mobility loss clearly or explore the psychosocial impact in detail.

Psychosocial wellbeing and independence are particularly important in this context; psychosocial wellbeing reflects the broader emotional and social consequences of PD, capturing changes in quality of life and identity over time,^35,36^ while independence reflects autonomy and functional capability, both of which can shape daily functioning and place a heavy burden on both people with PD and carers.^37,38^ Yet, despite their importance, current reviews do not provide a well-defined focus on mobility loss, a central aspect of PD, that underpins both psychosocial wellbeing and independence. Similarly, reviews examining the carer experience^19,39-41^ have not explored mobility-related challenges both for those with PD and carers. This leaves a clear gap for a focused synthesis that places mobility loss as the primary analytical lens.

Given the above, synthesising qualitative literature that explores how mobility loss affects independence and psychosocial wellbeing in people with PD and carers could offer valuable insights. This would address the current lack of a focused, in-depth exploration of mobility-related challenges in PD and provide a more comprehensive qualitative account that is currently missing. Therefore, this meta-ethnography aims to capture the impact of physical mobility loss on perceived independence and psychosocial wellbeing in people with PD and carers.

## Methods

This synthesis consists of a systematic review and a following meta-ethnography. Methods will be reported in accordance with the Preferred Reporting Items for Systematic Reviews and Meta-Analyses (PRISMA) and the reporting guidelines for meta-ethnography (eMERGe).^42,43^

### Phase 1: Getting Started

Meta-ethnography was selected as the most appropriate methodology because it allows both the interpretation and conceptualisation of how people experience a phenomenon and to build a theoretical understanding that extends beyond findings of individual studies, retaining the original interpretations within its analysis.^43-45^ This method distinguishes itself from other qualitative synthesis methods by re-interpreting authors’ conceptual constructs alongside primary participant data through a synthesis process, enabling the development of higher-order themes that extend beyond individual study findings.^46^ It is therefore particularly congruent with our focus on people with PD and carers, as it allows us to capture the complex and relational realities of mobility loss and its intrinsic link to the experience of PD. Our focus is on the lived experiences of PD and mobility loss from the perspective of the person with PD and carers. This method is well suited to synthesising lived experiences as it preserves the interpretive depth and contextual richness of qualitative studies.

### Phase 2: Deciding What is Relevant

Studies were included if they explored the lived experience of mobility loss in PD from the perspective of people with PD and/or the experiences of cares supporting a person with Parkinson’s-related mobility loss. The phenomenon of interest was defined as the impact of mobility loss on independence and/or psychosocial wellbeing in people with PD and carers. For the purpose of this review, mobility loss was considered as factors directly affecting freedom of movement, such as impaired walking, freezing of gait, balance difficulties, falls, and related challenges in physical movement. Eligible participants included people diagnosed with PD and carers of people with PD. Studies where mobility loss was attributable to conditions other than PD were excluded. Methodologically, qualitative studies and mixed-methods studies with a predominant qualitative focus were considered. Mixed-methods studies were included only when qualitative findings were reported separately. Exclusively quantitative studies, systematic reviews and meta-analyses were excluded. Only studies in written in the English language were considered.

A comprehensive search strategy was developed by the research team with no specialist involvement and was explicitly guided by the research question. Key concepts included Parkinson’s disease, caregiving, mobility impairment, psychosocial wellbeing, independence, and lived experience. Searchers used different variations of vocabulary to capture population, including combinations of Parkinson with caregiver, carer, and related variants. Additional search terms captured context such as mobility-related constructs (eg, mobility, gait, walking, combined with issues, impairment, or loss) and psychosocial outcomes (eg, psychosocial wellbeing, psychosocial functioning, independence, activities of daily living and quality of life). All qualitative study designs and methodologies were eligible, as were the qualitative components of mixed-methods studies provided that qualitative findings were reported distinctly. This included search terms such as qualitative, interview, focus group, mixed-methods, perception and lived experience.

A systematic search was then conducted by one reviewer (MH) across six health databases: Medline, PsychInfo, Embase, Web of Science, Scopus and CINAHL. The search was not limited to a specific timeframe and all relevant studies conducted up until November 2024 (ie, date of the search) were considered.

Methodological quality of eligible studies was assessed using the NICE quality appraisal checklist.^47^ This was done to assess conduct, content, utility of findings within each study, and to make the strength and weaknesses of the evidence based transparent. Appraisal was not used as a basis for exclusion but rather to provide a framework for interpreting findings. This approach was chosen because methodological limitations in reporting does not necessarily indicate low-quality research, and excluding studies based on strict appraisal criteria would have risked omitting valuable insights.^48^

### Phase 3: Reading Through the Studies

The eligibility of records was then assessed by two reviewers (MH and CW) in a title, abstract, and full-text screening. During title and abstract screening, studies were excluded if mobility loss was not related to PD, psychosocial wellbeing and/or independence, or if the focus lacked reference to lived experience. At full-text screening, studies were excluded if reference to mobility was limited, psychosocial outcomes and independence were examined solely through quantitative measures or were without relevant in-depth qualitative exploration, or where no link between mobility loss and psychosocial wellbeing or independence could be established. Reasons for exclusion were recorded, disagreements were resolved through discussion, and any conflicts were solved by an additional member of the reviewing team (KB). Key characteristics of each included study was recorded using a data extraction form by one reviewer (MH) and checked by another (CW; Table 1).

**Table 1.** Study Characteristics.

Author	Year	Aims/purpose	Location	Sample	Methods of data collection	Key findings related to review	Participant quotes	Interpretation of authors
Nazzal & Khalil	2016	To describe the daily living experiences and impact on lives of Jordanian individuals with PD	Jordan	8 people with PD, comprising of 5 men and 3 women, aged 60-80 years	Semi-structured interviews	Mobility and dependence on others Participants faced significant mobility issues that put a strain on both them and their carers. They often had to depend on family or friends for tasks like shopping and housework, even though they wanted to remain independent. While they appreciated the help, they still strongly desired to manage on their own. Example Quote: “The medication is really great, but it has restricted my life; I cannot go anywhere unless I have my tablets.” Impact on independence, family and household roles Relying on family for daily activities made participants feel they had less control over their own lives, which challenged their traditional roles at home. They valued family support but wanted to maintain as much independence as possible to feel good about themselves Stigma and social withdrawal Participants often faced staring, laughter and hurtful comments about their visible symptoms, like unusual walking patterns or tremors. This affected their self-esteem and led many to withdraw from social situations. Younger or recently diagnosed individuals felt this stigma more and often kept their condition private to avoid judgement. Example Quote: “Feeling that people are watching me, the stress, and the tension all together makes the symptoms get worse.” Emotional toll of living with PD Living with PD took a heavy emotional toll, leading to feelings of despair and exhaustion. They were worried about their future and found that while they needed medication, it also limited their freedom to move and act as they wanted. Example Quote:“I feel that I am in a state of continuous despair, stress, too much thinking. It is like living a nightmare.” Adaption and coping strategies Participants used various strategies to cope with everyday challenges, such as using scarves without buttons to make dressing easier. They found strength in accepting their condition early on, spiritual beliefs and support from family, which helped them adjust to life with PD. Many viewed their condition as a test of faith, providing comfort and resilience amid their daily struggles		The authors interpret stigma as a major influence on the lives of people with PD, leading to social withdrawal, emotional distress and heightened self-consciousness due to visible symptoms such as altered gait. This perception of being judged creates a cycle of avoidance and isolation, worsening psychological wellbeing Age differences emerged, younger participants (40s) reported greater disruption to life plans, professional roles and personal goals, with higher levels of depression and anxiety, while older participants found the diagnosis less disruptive The impact of PD was described as extensive and cumulative, with physical symptoms and emotional distress creating a “snowball effect” that limits daily activities, social connections and quality of life Family support was seen as both protective and problematic, reducing loneliness but risking overdependence and undermining independence. The authors highlight the complex nature of caregiving, where support can unintentionally reduce autonomy
Caap-Ahlgren et al.	2002	To explore women’s experiences of living with symptoms related to PD, and analyse how the symptoms influence their quality of life	Sweden	8 people with PD, all women, aged 63-80 years	Semi-structured interviews	Body image and visible symptoms Women with PD wanted to maintain a stable body image because they felt society accepted normal appearances. Symptoms like involuntary movements and changes in walking made daily life embarrassing and highlighted their differences Example quote: “Feel especially watched. People glance and wonder what is wrong” Impact on traditional female roles Participants found it hard to perform traditional female tasks like cooking and shopping, which impacted their independence and self-esteem. Not being able to do these tasks made them feel like they were failing in their roles. The women were worried about becoming passive and too dependent due to their difficulties in managing tasks. This struggle made them feel embarrassed and devalued Example Quote: “Doing household duties is troublesome. Most embarrassing is being unable to clean up” Declining physical abilities Participants noticed a decline in their physical abilities, leading to feelings of clumsiness and anxiety about daily activities. They could no longer meet their expectations. despair, stress, too much thinking. It is like living Example Quote:“Being clumsy is a handicap” Social participation and comfort in family settings Social interactions became tougher, with women feeling most comfortable with family, especially grandchildren, who did not judge them. Interacting with adults felt strained due to stigma and misunderstandings. Example quote:“Children are the best. They don’t ask as much as adults always do. With the grand-children I enjoy myself” Stigma, isolation and loss of identity Participants often felt a loss of social skills and connection. While family members were sympathetic, they often lacked true understanding, leaving the women feeling isolated. PD caused feelings of shame, especially in public. Women felt others viewed them as “incompetent,” leading to social withdrawal. They experienced a loss of confidence and identity because they could not contribute to their families or keep their previous roles. Example quote:“People around me think I am incompetent, that I am an idiot”		The authors interpret the experiences of women with PD as being shaped by the unpredictability of their physical and mental abilities, which disrupts traditional roles and erodes a valued sense of identity. Symptoms hinder their capacity to fulfil what they view as “typical female responsibilities”, creating fears of being perceived as passive or incompetent. This perceived loss of purpose undermines both their control within the home and their self-concept Social relationships are strained by a lack of understanding from family and friends. Although some express sympathy, frustration over fluctuating abilities leaves participants feeling misunderstood and less willing to engage socially, reinforcing their isolation. Stigma emerges as a central theme, with visible symptoms prompting fears of being judged as “incompetent” or “stupid.” in response, many avoid public situations to conceal difficulties The authors highlight the heavy emotional toll of keeping up appearances, which heightens shame and self-doubt. Participants express a strong desire to be seen as “normal” and capable yet increasingly avoid tasks such as shopping due to their complexity. This retreat from once routine activities symbolises a profound loss of independence and self-sufficiency, marking a painful shift from their previous lives and aspirations
Davey et al.	2004	To explore views and experiences of the informal carers of repeat fallers with PD	UK	14 family carers, comprising of 11 women and 3 men, aged 44-79 years	Semi-structured interviews	Falls and risk-taking Falls often occurred due to symptoms like leaning forward, freezing during movement and difficulty turning. The desire for independence led individuals with PD to take risks, which increased the chances of falling. As a result, they experienced a significant loss of confidence and grew more afraid of falling, further limiting their social activities and increasing isolation at home. Many carers injured themselves trying to catch or lift their spouse. Most carers had no formal instruction on how to manage falls or help someone get up safely, and they didn’t know where to get this information Carer vigilance Carers were constantly anxious about falls and their impact on their loved one’s quality of life. To prevent falls, carers stayed vigilant, rarely leaving their partner alone and provided advice on posture and walking. They also made changes to the environment, such as installing grab rails and using equipment to reduce fall risk. Example Quote: “He would fall everyday if I wasn’t vigilant, but I have to make sure I’m with him when he tries to go to the lift, or tries to go to the toilet, or anything on his own. I Have to make sure I’m with him to help him, to steady him, as he falls down when he tries to do it on his own.” Psychological and emotional impact on carer Falls had a profound psychological and social impact on carers, causing anxiety, worry, fear, anger and frustration. Many carers felt their freedom was limited because they avoided leaving the person with PD alone and rushed through their personal activities. This constant responsibility affected their physical and emotional wellbeing. Example Quote: “I never like to leave him on his own because if he falls no one’s here to help him. . . I Have done it but very rarely and very much with a feeling of, you know, sort of resisting the idea of leaving him on his own…I go out and I do everything I have to do as quick as possible.” Impact on social participation and stigma The fear of falling also impacted social activities outside the home, causing carers and individuals with PD to avoid outings. This led to feelings of social isolation and stigma due to the limitations imposed by the condition		The authors highlight the central role of informal carers in ensuring the safety and well-being of spouses with PD, particularly in preventing falls. This responsibility places significant pressure on carers, limiting their freedom, reducing attention to their own needs, and contributing to emotional strain and social isolation. Carers often develop fall-management strategies based on personal experience, but the lack of formal training or support adds to their stress and isolation. The authors emphasise the need for structured guidance and support to reduce caregiver burden and improve outcomes for both parties
Hammarlund et al.	2018	To explore the overall impact of living with PD	Sweden	19 people with PD, comprising of 11 women and 8 men, aged 55-84 years	Semi-structured interviews	Declining physical and mental abilities As their physical and mental abilities declined, managing daily tasks became more difficult. Simple activities like walking turned challenging due to slowed movement, stiffness, and sometimes an inability to move at all. Mood swings and feelings of panic added to their struggles, making them anxious about the future and losing their independence. Some participants felt depressed and helpless, questioning why they should continue living given their limitations. Example Quote: “I couldn’t walk any further than maybe out of the house and into the garden…then I found myself in a world that was totally my own…I became completely stiff…my wife shouted at me that “you have to move”, but I was completely stuck.” Increased dependence and changing family dynamics As PD progressed, participants became more dependent on others for personal care and daily tasks. This increased reliance changed family dynamics and led to a profound sense of lost identity. Many felt bitterness and shame, believing that their physical and mental changes had taken away their former selves. They tried to hide their condition, feeling belittled, insecure, and like failures, which diminished their confidence. Example Quote:“Although I have Parkinson’s disease, I want to be respected. I Want to be able to do…take care of things like I always did in the past and I want to take an active part in society…and I don’t want to be looked down upon in any way…I want to be accepted even though I do have Parkinson’s.” Social withdrawal and isolation Their condition limited their ability to engage in social activities, leading to feelings of isolation. Many participants felt embarrassed by their changed appearance and chose to withdraw from social situations to avoid discomfort. This withdrawal increased their loneliness and shame Stigma, judgement and loss of self-worth Participants felt a loss of self-worth based on how others viewed them. They experienced stares, rude comments, and judgment, which reinforced their feelings of shame. They longed to be recognized as their true selves, rather than as a burden or someone incapable Practical and psychological coping strategies Participants used multiple aids, eg, alarm devices, adapted chairs, wheelchairs, and modified activities, eg, showering instead of bathing or avoiding certain tasks to prevent falls. Views on walking aids were mixed; they were supportive for some and stigmatising for others. Participants also planned activities in detail to manage unpredictability, maintained positive attitudes, and used downward comparisons with those worse off		The authors interpret PD as a profound life change that disrupts daily functioning, social relationships, and self-identity. Drawing on self-determination theory, they link reduced autonomy, competence and social connection to diminished self-worth and increased isolation. Physical symptoms, mood changes, and depression intensify this loss of control, creating a sustained psychological burden. They note that inability to participate in valued social and physical activities erodes confidence and reinforces feelings of incapacity. Coping strategies are described as essential to preserving independence, eg, the use of assistive devices, environmental adjustments, and emotional regulation techniques, enabling participants to reclaim some control despite the progressive challenges of PD
Johansson et al.	2019	Explore the meaning of balance for people with PD and the beliefs they hold regarding their ability to influence their balance in everyday life	Sweden	18 people with PD, comprising of 8 women and 10 men, aged 46-83 years	Semi-structured interviews	Emotional impact of reduced balance Participants felt a strong emotional impact from their reduced balance, which often forced them to change or give up activities they once did easily. Slower walking and the need to concentrate to avoid falling diminished their sense of freedom and independence, leading them to adopt new ways of managing their condition. Example Quote:“You lose the freedom to move in the way that you would like to. That must be the biggest freedom that you have, to be able to go where you like” Resilience and maintaining social identity Despite balance challenges, participants remained determined to engage in activities with friends and family. They showed resilience by not allowing PD and balance issues to define their social identity or relationships. Example Quote:“I feel now that I have accepted the disease and that I am comfortable to be in certain environments where I don’t really have control.” Balance is both physical and mental Participants viewed balance as both a physical and mental issue. They described how an “off-period” impacted them physically, emotionally, and mentally. For some, balance was seen as a state of ease that allowed movement without constant thought Unpredictability and strategies for coping The unpredictable nature of PD, like sudden freezing episodes, forced participants to be extra careful, such as walking in the middle of train platforms. Concentration was essential for safely performing tasks that required balance. To cope, they used strategies like accepting reduced control, not feeling sorry for themselves, distracting themselves with physical activity, and sticking to past routines to feel normal		The authors interpret the fluctuating symptoms and restricted freedom in PD as drivers of unpredictability and loss of control in daily life. This instability undermines participants’ ability to manage their condition with confidence. They note that coping relies on a combination of mental resilience and practical strategies, such as sustained focus and determination to maintain balance, reflecting a strong motivation to preserve independence. These coping methods combine physical adjustments with mental strength, highlighting the participants’ commitment to keeping their independence and sense of self, even as their condition brings new challenges
Huang et al.	2022	To explore how perceptions of fall risk influence decisions to undertake activities in people with PD who have fallen, along with their care partners	Australia	8 people with PD, comprising of 6 men and 2 women, aged approx. 60-90 (age group) 8 family carers, comprising of 6 women and 2 men	Semi-structured interviews	Emotional impact and loss of independence Participants were emotionally affected by their poor balance, which often forced them to change or stop activities they used to do easily. Walking slower and needing to concentrate to avoid falls made them feel less free and independent, so they found new ways to manage their condition. For carers, it was frustrating to see their partner resist using mobility aids that could improve safety. Both described feelings of embarrassment and frustration at needing help after a fall. Carers described anxiety when they knew their partner was moving around, especially I public spaces. Example Quote:“I get cross that she doesn’t use the walker. I Try to get her to use it all the time… so just trying to teach her to rely on it, I suppose it’s a frustration for us.” “I just feel so stupid. Had a fall and not being able to get up and having to rely on someone to come and do it… Just make a nuisance of myself” Resilience and maintaining social life Despite their balance challenges, participants were determined to spend time with friends and family. Many refused to let PD and balance problems define their social lives, but for some fall risk meant they were unable to participate or were just left out. Carers often influences what activities were done and sometimes accompany the person with PD to maintain safety Example Quote:“I can’t do other things that she (a friend) does. She’s joined Probus…she would have invited me if I didn’t have Parkinson’s…I’m upset about it” Balance as a physical and mental challenge Participants saw balance as both a physical and mental challenge. They noted how “off-periods” affected them physically, emotionally and mentally. For some, balance felt like an easy automatic state that let them move without constant thought. Carers described watching for signs of fatigue or freezing and prompting the person with PD to slow down or focus when walking Coping with unpredictability Because PD is unpredictable, with sudden freezing episodes, participants had to be extra careful, like walking in the middle of train platforms. Concentration was key for safely doing tasks that required balance. To cope, they accepted their limitations, distracted themselves with physical activities, and stuck to familiar routines to feel normal. Carers often positioned themselves to stead or catch their partner if needed, and some limited time they spent away from home		The authors interpret participants’ decision-making as a constant negotiation between safety and independence, a tension common in older adults but particularly pronounced in PD. This negotiation involved both people with PD and their carers, with carers often acting as “risk filters” who influenced, supported, or sometimes restricted certain activities in the interest of safety. Choices to avoid, adapt, or seek assistance were shaped not only by the risk of falling but also by a strong emotional need to preserve autonomy, for both partners, as carers also valued their own independence. Past experiences with falls and heightened awareness of vulnerability were seen as powerful influences on daily behaviour, guiding carers’ vigilance and the person with PD’s risk-taking or avoidance. Consistent with broader research, the authors note that individuals often continue engaging in activities despite fear of falling, using adaptation to maintain normalcy and identity. For carers, this required balancing protective instincts with respect for the person’s autonomy. This interplay between caution and autonomy underscores the complexity of PD care, where independence is pursued alongside an ongoing recalibration of risk in the context of a shared daily life
LaGrone et al.	2020	To explore how deficits in balance control manifest in everyday life and how it is managed by people with PD	Sweden	18 people with PD, comprising of 9 men and 9 women, aged 46-83 years	Semi-structured interviews	Use of aids and support Participants had different needs for walking aids, from using walkers to holding onto objects around them, based on how stable their balance was. While some needed no support, many adjusted their movement to feel secure, often using aids to help prevent falls. Some mentioned that walkers required constant attention, which could make walking harder Impact of mobility loss on daily life PD increasingly limited participants’ freedom, making them change how they managed daily tasks. Activities that used to be simple now required careful planning and adjustments to deal with balance issues. Fluctuating mobility and “off” periods created additional uncertainty, with participants planning tasks around medication and “on” periods to avoid instability and freezing Restrictions on social participation and independence Reduced mobility affected participants’ ability to make plans or attend social events, leading them to stay home more or rely on help from others. This loss of spontaneity made them feel restricted, which impacted their mood and sense of independence. Activities like going to town or socialising were greatly reduced, causing frustration. Crowded public spaces, narrow environments, and public transport were particularly challenging, often prompting avoidance. Example Quote:“I was free to go to the store and try on clothes and shows. Go and look at something, an exhibition or concert. Meet friends and go to the movies or organise something and go together but now it is…” Coping strategies for unpredictability To handle unpredictability, participants used strategies like doing one task at a time, using adaptive equipment, or asking for help during difficult times. They broke activities into smaller steps, consciously focus on each movement and postponed tasks to more stable times of day. Environmental adaptations, such as rearranging furniture or avoiding certain surfaces, helped reduce fall risk. Careful planning and movements helped them manage daily tasks better, although these strategies often reminded them of their limitations. Example Quote: “I plan how I will walk”		The authors interpret the adaptive strategies developed by people with PD as a reflection of both mistrust in their body’s reliability and a strong desire to preserve independence. Consistent with previous research, they note that balance problems, freezing of gait and fear of falling drive avoidance or reduction of motor and social activities, narrowing personal freedom and contributing to emotional distress, isolation and dependence. Participants reported that basic self-care tasks often required intense concentration to perform safely. While mobility aids were essential for support, they could also hinder task performance, underscoring a complex relationship between assistance and self-efficacy. The authors suggest that a deeper understanding of these experiences could inform more tailored therapeutic approaches that respect the need for autonomy in daily life
Owen et al.	2022	To explore how people with PD and their family carers understood and experienced falling and health care services relating to fall prevention and management	UK	20 people with PD, comprising of 14 men and 6 women, aged 57-85 18 family cares, comprising of 5 men and 13 women	Semi-structured interviews	Emotional impact of falls Both people with PD and their carers felt loss, frustration and isolation due to falls. Carers often expressed these feelings more than the individuals with PD. Some participants associated medical equipment, like walking aids, with aging, making them hesitant to use it. Over time, some dyads developed acceptance or normalised falls, while others used humour, distraction or peer support to cope. Example Quote: “If, heavens above, I have to go into a wheelchair or something like that the things are already in place, which I resent slightly, but I realise that you know, it has to be done.” Coping with fear of falling To deal with their fear of falling, participants developed coping strategies, mainly seeking support from each other. Some people with PD found it hard to socialise outside their close relationships due to friends not understanding PD, while others avoided certain activities entirely. Joining local PD or carer groups provided understanding and reassurance. Carers described being constantly on edge, especially when their partner was moving around, and felt more at ease when someone else was there to supervise. Example Quote:“I’m on edge all the time wondering…is he going to fall? We have a sitter twice a week and I feel happy going out…otherwise, I manage all the time.” Practical strategies and mobility aids Carers often helped manage freezing episodes by ensuring medication was taken on time, using specific cues and providing physical assistance when needed. They also made practical changes at home, such as keeping doors open and reducing clutter. Mobility aids, chosen based on the environment and symptom severity, helped people with PD feel safe and independent, allowing them to participate in activities outside the home. Some aids, however, were viewed as stigmatising or a visible sign of disease progression Impact on carers freedom Worries about people with PD falling when alone made carers limit their own outings, sometimes altering work schedules or avoiding going out entirely. Others timed their activities for when the person with PD was asleep or occupied. This restriction contributed to feelings of lost personal freedom and a sense of always being ‘on call’. Example Quote:“I don’t feel that I’m in control of my time. It’s like almost having a permanent, a full-time job.” Changing relationship dynamics The relationship dynamics often shifted due to falls and prevention needs. Carers took on more household tasks, which sometimes made people with PD uncomfortable or dissatisfied. Some carers felt that their partner had become too dependent on them. Despite these challenges, some couples found positive changes as they adapted and discovered new activities together Carer identity and role strain Carers expressed distress about the shift from being a partner or friend to a primary carer. They described the role strain from helping after a fall, constantly adjusting routines, and restricting their own activities. Balancing safety responsibilities with maintaining their personal identity and the original nature of their relationship was a constant challenge. Example Quote:“I like to think of myself as the husband…it is difficult…I don’t like the term carer.”		The authors interpret falls as a pivotal event that reshapes the lives of both people with PD and their carers, disrupting daily routines, independence and social participation. Carers often limit activities to reduce fall risk, a strategy that can unintentionally contribute to deconditioning or heightened fear of falling. They note that carers develop personal coping strategies to manage the ongoing threat of falls, which may support emotional wellbeing but also increase their own physical risk, particularly during manual assistance. A reluctance to involve emergency health professionals leaves carers assuming full responsibility for falls management, frequently without adequate support, leading to both physical strain and emotional stress
Parry et al.	2022	To examine the experience of people with PD when walking in different social situations and improve understanding of how this affects participation in meaningful activities	Paris, France	14 people with PD, comprising of 11 men and 3 women, aged 30-69	Semi-structured interviews and observations	Emotional impact of being observed Participants, especially those experiencing shuffling or freezing, felt very self-conscious about how others looked at them. Hurtful glances, expressions or laughter intensified discomfort in public and could even worsen symptoms, such as triggering or prolonging freezing episodes Example Quote:“People staring… I try not to pay too much attention, but I can still feel them looking. It hurts all the same” Impact of public perception on mobility Concerns about appearance affected participants’ walking in public. Some adjusted their gait or posture to appear more ‘normal’, while others chose aids that would signal a medical condition more clearly. Participants described feeling more at ease in private or familiar settings, where scrutiny was lower, and more tense in public spaces, particularly crowded or confided ones Strategies for managing social perception Participants used various approaches to influence how they were perceived, including visible mobility aids, altering gait, explaining their condition or avoiding certain environments altogether. These actions were aimed at either masking mobility problems to appear healthy or legitimising gait difficulties by making their cause clear. Supportive interactions, especially with people who understood PD, reduced self-consciousness and, in some cases, improved walking performance		The authors interpret the management of public perception as central to the social experience of people with PD, with considerable effort directed toward maintaining a positive self-image. Strategies include altering posture or gait, selectively sharing information about PD, and avoiding social situations to reduce the risk of misunderstanding. Drawing on Goffman’s theory of social interaction, they suggest that stigma arises when visible symptoms, particularly walking difficulties, lead others to misinterpret them as rude, disabled or intoxicated, provoking feelings of shame. Referencing soundy et al.’s review, they note that changes in appearance can disrupt social connections, creating a gap between self-perception and public perception. This disconnect reinforces the importance of gait and physical presentation in sustaining identity and belonging, underscoring the complex interplay between mobility, stigma and self-image in PD

*PD = parkinson’s disease.

Key: Yellow shading indicates studies involving people with PD, blue indicates studies involving carers, and green indicates studies involving both.

### Phase 4 and 5: Determining How Studies Are Related and Translating the Studies Into One Another

We aimed to establish how the included studies are related by systematically comparing their findings. One reviewer (MH) identified first-order constructs (ie, direct participant quotes) and second-order constructs (authors’ interpretation). This involved exploring and grouping together recurring themes and perspectives that emerged across studies, focusing on mobility loss and its impact on people with PD and carers. The emerging constructs were then discussed within the wider team to support triangulation, enhance credibility and ensure rigour in interpretation. Once we identified concepts that aligned with each other, we integrated findings to create a broader, shared understanding of mobility loss on PD. Where studies presented differing or contradictory interpretations, the inconsistencies were explored, and we included a more nuanced understanding of the phenomenon.

### Phase 6: Synthesising Translations and Expressing the Synthesis

After translating the studies, MH synthesised the key constructs to create third-order constructs, with iterative review and feedback from the wider research team, that represented new interpretations that extend beyond the original findings. This phase focused on detailing the varying perspectives, both from people with PD and carers, to generate a shared understanding of the physical, emotional, and social effects of mobility loss. We identified overarching themes which are articulated below in a comprehensive account of the impact of mobility loss in PD.

## Results

Following database search, 841 records were identified. Nine qualitative studies were included in the meta-ethnography, following the screening process (see Figure 1). Regarding methodological quality, the included studies were deemed appropriate (see Appendix A).

**Figure 1.**
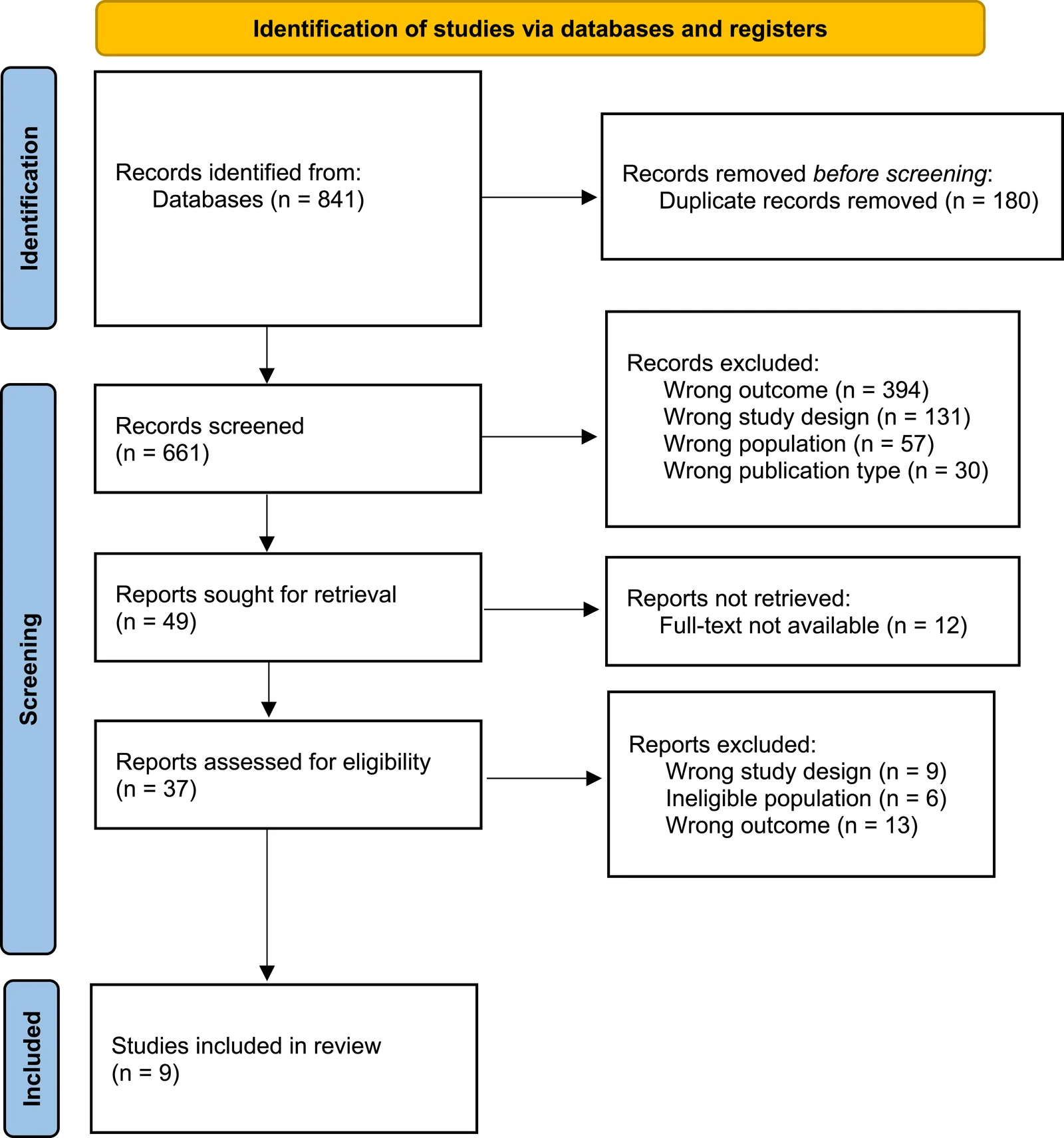
PRISMA flow diagram.

All studies covered the experience of mobility in PD, and three included perspectives from informal carers. Among the 153 participants, 113 were people with PD and 40 were family carers, ages ranged from 30-90 years, with an equal representation of men (48%) and women (52%). Ethnicity was scarcely reported; most represented primarily Western or European populations, with one study focusing on a Middle Eastern (Jordanian) group. See Table 1 for further details.

### Analysis of Findings

Table 1 outlines the key characteristics and findings of the included studies. Five overarching themes emerged, describing the impact of mobility loss on both people with PD and carers. See Table 2 for a summary of findings.

**Table 2.** Summary of Findings.

Themes	Subthemes	Findings
Findings for people with Parkinson’s disease		
Struggle for independence and associated self-esteem	• Struggle for independence	• Decline in physical capability affects confidence in performing daily activities
	• Reduced independence	• Increased reliance on assistance leads to feelings of incompetence and distress
	• Dependence on others	• Loss of independence particularly impacts women’s self-esteem and identity
	• Diminished self-confidence	
	• Reduced physical capability	
	• Restrictions of personal freedom	
	• Loss of identity	
	• Difficulties with balance and fall risk	
Navigating personal relationships	• Limited social interaction beyond family	• Social interactions are strained; friends often lack understanding of mobility challenges
	• Difficulty participating in social activities	• Family support is crucial for adapting to mobility issues, but the desire for autonomy and independence remains
	• Importance of family support	
	• Resilience in maintaining social engagement	
Perceived stigmatisation and social isolation	• Perceptions of negative judgment	• Fear of stigma and negative public reactions prompts social withdrawal
	• Fear of being stigmatised	• Visible symptoms and embarrassment lead some to avoid social situations, increasing isolation
	• Embarrassment related to visible physical limitations	
	• Reluctance to use mobility aids	
	• Impact of unpredictable symptoms on social participation	
Resilience and adaptation	Psychological impact	• Anxiety, depression and concerns about future decline are common as mobility worsens
	• Anxiety and low mood	
	• Fear and concerns about the future	
	• Impact on self-esteem and personal identity	
	Adaptations to living with PD	• Physical adaptations: Use of mobility aids, environmental modifications and activity planning to maintain safety and independence
	• Practical physical adaptations	• Psychological adaptations: Emotional support from family and coping strategies such as acceptance, positive outlook and connection to support networks
	• Psychological adjustments	
Findings for carers		
Impact of mobility on carer wellbeing	• Physical strain and vulnerability	• Significant physical and emotional strain from providing mobility-related support
	• Need for constant vigilance	• Constant vigilance to prevent falls leads to anxiety, social isolation and restricted personal freedom
	• Limitations on personal freedom	• Female carers often experience physical vulnerability when assisting with mobility
	• Emotional burden	
	• Shifts in role changes and identity	
	• Frustration and resentment	
	• Resilience and emotional connection	

#### Findings for People With PD

##### The Struggle for Independence and Associated Self-Esteem

Across studies, the impact of mobility loss on diminished independence emerged as a central theme.^49-56^ As mobility declined, participants expressed a growing lack of confidence in their ability to perform even basic activities of daily living, which directly affected their sense of independence. This decline in physical capability often led to feelings of incompetence and emotional distress.^54,56^ The reliance on assistance for routine tasks was frequently experienced as a direct challenge to their identity and self-perception as capable people.^52^

As participants with PD faced increasing difficulty with functional movements such as walking, standing, and moving around the house, household and self-care responsibilities gradually shifted to family members.^53^ Mobility limitations were frequently cited as the main reason for this shift, as participants grew to distrust their body’s reliability, leading to feelings of restricted freedom and a loss of identity, especially when mobility aids or assistance became necessary for safety.^50,52,56^ Impaired balance and the associated risk of falls also led people with PD to modify or avoid activities which, though necessary for safety, highlighted the progressive nature of their condition and diminished their sense of autonomy.^50,51^ This loss of independence was especially challenging for women, as relinquishing control over household tasks affected their self-esteem and self-worth.^54^ This challenged their sense of femininity, as they could no longer maintain the household or their physical image in the way they once had.

The desire to maintain independence sometimes led participants to take risks, such as attempting to perform certain activities without assistance. While this may have temporarily boosted self-esteem, it also increased the risk of falls, posing both physical and emotional risk such as feelings of helplessness and dependency.^56^ Conversely, in other cases, the fear of falling resulted in a reluctance to engage in physical activities altogether, whether by personal choice or following advice from their carer. This fear further intensified participants dependency on others,^49,55,56^ creating a cyclic relationship between mobility loss, dependence, and reduced self-esteem.

##### Navigating Personal Relationships

Social interactions, particularly beyond the immediate family, were often limited. Some participants mentioned that their friends lacked an understanding of the physical challenges posed by PD, which made socialising difficult and strained friendships.^49,54^ However, for many, family support was instrumental in helping them adapt to their mobility challenges.^53^ That said, the desire to maintain independence persisted; participants tried to balance between seeking support while striving for autonomy.^53^ This balancing act was complex, as mobility impairments often fluctuated, causing frequent adjustments in the level of support needed.

Despite these difficulties, people with PD showed resilience in their efforts to engage socially.^51^ Some found that the emotional support provided by family and friends could temporarily ease their mobility difficulties, such as reducing anxiety during social interactions, which in turn improved their gait and confidence in their mobility.^57^

##### Perceived Stigmatisation and Social Isolation

Mobility impairments in PD often lead people with PD to feel perceived differently by society due to visible symptoms such as impaired gait, tremors, and other motor dysfunctions. This fear of stigma was a recurring theme, with many participants reporting social withdrawal and psychological distress due to the public’s reaction to their symptoms, such as staring, laughing, or hurtful comments.^50,52-57^ Embarrassment over their physical limitations led some participants to avoid social situations, fearing judgment or misunderstanding, while others actively tried to conceal their condition or avoided mobility aids like walking sticks, despite the increased fall risk.^51,57^ The effort to manage how others perceived their mobility became a constant challenge, with some participants adopting strategies such as using a cane to ‘legitimise’ their walking problems,^50,57^ which they found emotionally difficult to accept. For many, the stigma attached to these visible symptoms was isolating, with participants describing how being seen as “incompetent” or even “like an idiot” diminished their self-worth. Some even adjusted their posture or “gait image” to manage perceptions yet still struggled with feelings of shame and a loss of identity.^54^

The unpredictability of symptoms, such as the sudden shift between “on” and “off” periods, further complicated social participation. People with PD worried about the possibility of freezing or falling in public, which often left them feeling excluded or isolated.^54^ This led many to avoid social events altogether, creating a cycle of isolation that had a profound impact on the psychosocial wellbeing.

##### Resilience and Adaptation

###### Psychological Impact

People with PD frequently reported heightened anxiety, depression, and a persistent sense of loss as their mobility declined. The physical limitations imposed by PD often led to significant psychological toll, with concerns primarily focused on the progression of the condition, the physical deterioration comes with it and worries about what the future may hold.^52^ The fear of losing the ability to walk independently, the increased risk of falls, and the dependency on others for basic mobility-related tasks contributed to feelings of helplessness and despair.

For many participants, their self-esteem and sense of identity were closely tied to their ability to move independently, and the gradual loss of this ability amplified feelings of anxiety and depression.^50,53^ Mobility impairments were a constant reminder of the progressive nature of PD, leading to persistent worries about how their condition would affect their future quality of life, ability to engage in social activities, and maintain independence.^50-53^

###### Adaptations to Living With PD

For people with PD, developing adaptation strategies was essential for maintaining a sense of independence and quality of life, especially amid increasing mobility challenges. These adaptations were both physical and cognitive, designed to help manage with daily activities and minimise the risk of falls, while also addressing the emotional challenges that come with the progressive decline in mobility.

## Physical Adaptations

Mobility aids, such as walking sticks, walkers, and wheelchairs were commonly used by people with PD to assist with daily activities and provide stability during movement.^50,52,57^ These aids were vital for preventing falls and maintaining a level of independence, especially when navigating uneven terrain or crowded spaces.^49^ Additionally, people with PD would often plan their activities around their “on” periods, when their mobility was less restricted due to the effects of the medication, ensuring that more physically demanding tasks were done when their movement was more reliable.^50,51^ For many people with PD, modifying the environment, such as rearranging furniture to create clear walking paths or installing grab rails, was another important strategy to help reduce the risk of falls within the home.^49,55^

However, while these physical adaptations were necessary for safety and functionality, they also served as a reminder of the progressive nature of PD. The constant need for aids and adjustment often reinforced the reality of declining physical abilities.^56^ For some people with PD, using mobility aids, although beneficial, was perceived negatively because it signalled their disability, impacting their self-esteem and psychological wellbeing.^49,52,56^ This strain between the need for physical assistance and the desire for independence was a recurring emotional challenge for people with PD.

### Psychological Adaptations

To cope with the emotional toll of mobility loss, people with PD often adopted various psychological strategies. Maintaining a positive outlook and accepting their physical limitations were key factors in preserving emotional resilience.^52^ People with PD who were able to find comfort in spirituality, family support, or social networks were better equipped to manage the psychological impact of their condition.^53^ The involvement of family members, who often provided both emotional and practical support, played a crucial role in helping people with PD navigate daily life, even as they sought to maintain their independence.^53,56^

For some participants, continuing to engage in physical activities that were once part of their daily routine, albeit adapted, provided a sense of normalcy, and helped counteract feelings of helplessness.^57^ These adaptations allowed them to continue participating in meaningful activities while minimising the risk of falls. However, despite these adjustments, many people with PD still experienced ongoing frustration and emotional distress due to their progressive mobility impairments.^52^

#### Findings for Carers

##### Impact of Mobility on Carer Wellbeing

Mobility impairments in people with PD significantly increase the burden on carers, creating both physical and emotional strain. The physical demands of assisting their care partner, particularly in preventing or responding to falls, place carers under immense pressure. Many carers felt they had to remain constantly vigilant, always on alert to prevent falls, which created ongoing anxiety and worry.^55^ This constant state of alertness restricted their own personal outings, contributing to a sense of loss and isolation, as carers often felt they could not possibly leave the person with PD one alone for extended periods due to fear of falls.^49,56^

Falls or the risk of falling were a primary concern. Carers, especially female carers, reported feeling physically vulnerable when trying to assist their care partner, particularly after a fall. The task of lifting or supporting someone with impaired mobility often felt overwhelming, and some feared injuring themselves in the process.^49^ This concern was further compounded by the unpredictable nature of PD symptoms, such as freezing episodes, which often necessitated immediate physical assistance, placing both the carer and the person with PD at risk of injury.^55^

The emotional toll of this constant vigilance was significant. Carers frequently expressed feelings of exhaustion and emotional depletion, worsened by the lack of personal time or breaks from their caregiving role. Adjusting daily activities to prevent falls, such as modifying the home environment by keeping doors open or reducing clutter, became routine but also deepened the sense of responsibility.^49^ While these adaptations were necessary, they often reinforced feelings of dependency for the person with PD and heighted the emotional burden for the carer. Carers experienced frustration and isolation as their social activities and daily routines were increasingly shaped by the need to manage mobility-related challenges.^56^

This constant management of mobility impairments not only reshaped daily activities but also strained the emotional dynamics within the dyadic relationship. For many carers, the increasing physical demands of assisting with mobility, such as helping with walking or preventing falls, reshaped their sense of identity, making them feel defined by their caregiving role. As they took on more domestic responsibilities and focused on the mobility needs of the people with PD, carers often experienced feelings of resentment, particularly when they perceived that the person with PD was overly dependent on them.^49^ Despite the challenges, some carers found moments of resilience and emotional connection, especially when adapting activities to maintain a sense of normalcy and shared time together.^49^ However, these positive moments were less frequently reported and were dominated by the overwhelming demands of caregiving.

#### Comparison of Experiences Between People With PD and Carers

The experiences of people with PD and carers showed notable commonalities as well as important difference, reflecting the interconnected yet distinct ways mobility challenges shape daily life. These relationships are brought together in Figure 2, which exhibits a model of mobility-related impact in PD integrating the perspectives of both groups into a single conceptual framework. In this model, people with PD and carers experience shared impacts, such as loss of independence, emotional strain, social isolation, adaptation and resilience. This also highlights the reciprocal influence between both groups; for example, as people with PD lose independence through mobility loss, carers take on greater responsibility, which could then restrict the autonomy of the person with PD.

**Figure 2.**
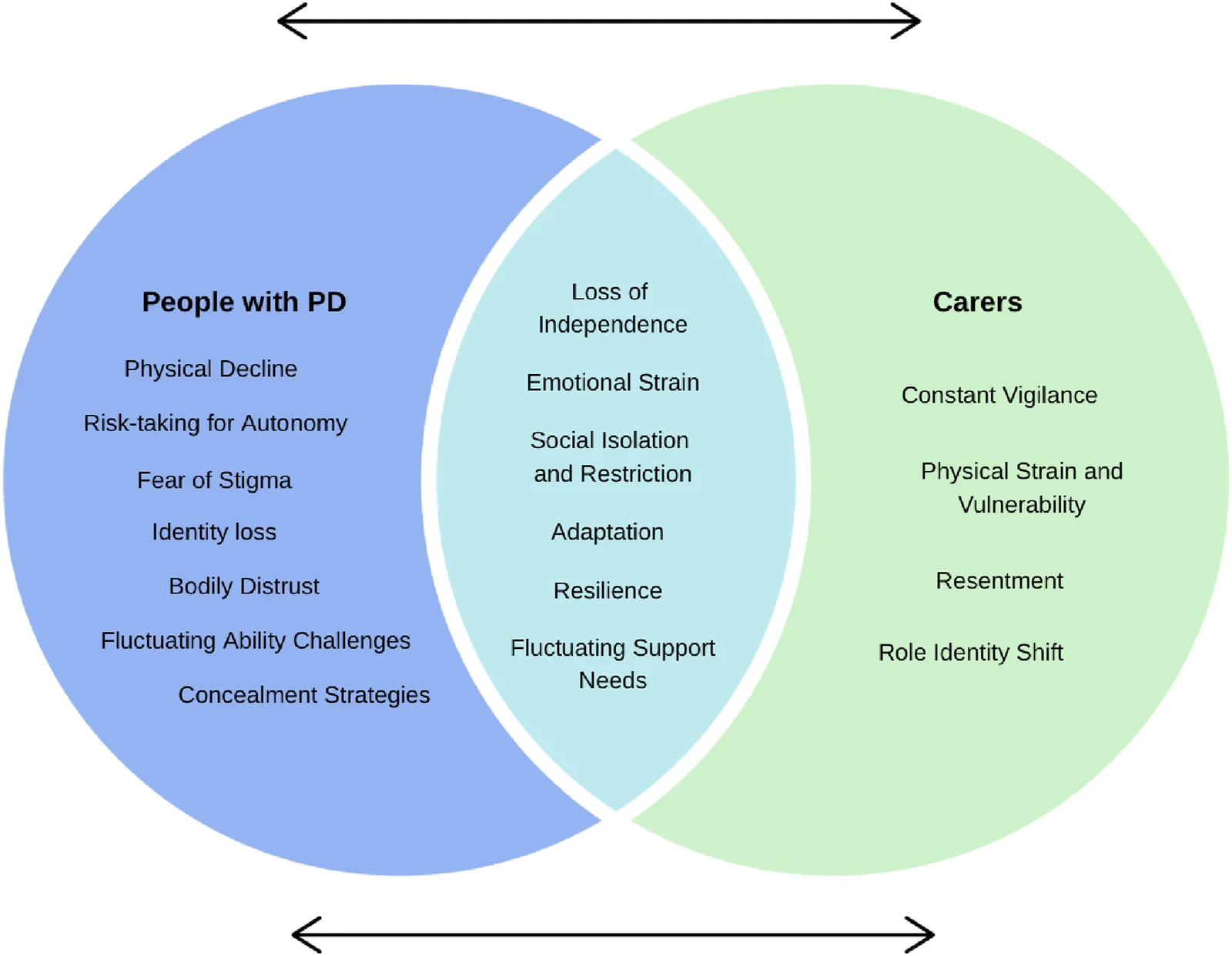
Dyadic model of mobility-related impact in PD

Despite these commonalities, the underlying source and how they were experienced varied. For people with PD, the impact stemmed primarily from mobility loss, fear of stigma, and the tension between maintaining autonomy and managing safety. Whereas for carers, the strain was caused by constant vigilance, physical demands, and a shift in role identity. Risk was also perceived differently, people with PD sometimes took physical risks to preserve their autonomy, while carers were more likely to limit activities to reduce fall risk. Gendered patterns emerged in both groups; women with PD often linked mobility loss to the loss of traditional household roles and self-image, and female carers described physical vulnerability when providing assistance with mobility.

Although a refutational synthesis identified few direct contradictions across participants, some differences emerged within the accounts of people with PD. These included responses to fall risk, with some participants taking physical risks to preserve independence and self-esteem, while others avoided certain activities altogether due to fear of falling or advice from carers. Perception of mobility aids also varied, as some participants viewed them as tools to maintain safety and independence, while others perceived them as stigmatising symbols of disability which negatively affected self-image. Approaches to social participation also slightly differed. Many withdrew completely from social settings due to embarrassment or stigma, whereas others remained active in the community, but often making efforts to conceal their symptoms. Similarly, experiences were quite consistent across carers, however some differences still emerged. The experience of physical strain only came up with female carers, they described feelings of physical vulnerability when assisting with mobility or after a fall, while others focused more on emotional or practical challenges. Emotional responses to the carers’ role also differed, with some expressing frustration, exhaustion or resentment, while a few focused on resilience and emotional connection. So, while the overarching impacts of mobility change are widely shared, the ways in which people experience and respond to these challenges can differ considerably.

## Discussion

This meta-ethnography is the first to synthesise literature that provides a detailed account of the impact of mobility loss in people with PD and the experiences of carers managing the associated physical and emotional challenges. Across studies, findings consistently emphasised that mobility loss has significant consequences for both the independence and psychosocial wellbeing in people with PD. Common themes included loss of autonomy, increased reliance on carers, and the emotional strain associated with mobility loss. These findings echo earlier research linking reduced mobility to diminished quality of life and increased emotional distress in people with PD, due to the growing dependency on others for everyday activities.^58,59^ However, our synthesis extends existing knowledge by integrating the perspectives of carers, who described constant vigilance, physical strain and restrictions to their own independence as key consequences of supporting a person with mobility loss. Previous studies have found that the carers’ psychosocial wellbeing is associated with the person with PD’s ability to remain mobile.^60^ Studies examining the caregiving experience in PD also highlight the significant influence of non-motor symptoms, particularly cognitive impairment and the burden of treatment on carer wellbeing.^61^ Taken together, these findings highlight mobility loss as a shared challenge; however, less is known qualitatively about how mobility is experienced by these groups and how it impacts the quality of life of both the person with PD and the carer.

The unpredictability of PD symptoms, such as the sudden shift between “on” and “off” periods, emerged as a persistent challenge that complicated social participation and highlighted a distinctive aspect of PD compared with other progressive conditions. This unpredictability is evident throughout the themes, impacting both the person with PD and the carer. For people with PD, abrupt mobility changes made it challenging to engage in social and everyday activities with confidence, often contributing to social withdrawal. For carers, fluctuating care demands required continuous adaptation and resulted in cumulative physical and emotional burden. These findings underline the need for flexible, dyadic care models that challenge traditional patient-centred approaches and adapt to the unpredictable nature of PD whilst also supporting both people with PD and their carers. Models that recognise the person with PD and the carer as an interconnected unit in which changes in mobility directly influence carer burden, emotional wellbeing, physical health and social participation.^62^ And further, care models that extend beyond physical symptom management to incorporating more holistic support.^62,63^ This would require health care professionals to acknowledge reciprocal impacts within the dyad, support people with PD to remain socially engaged despite mobility fluctuations and also recognise carers as care recipients alongside patients within care planning. To capture these interlinked experiences, we developed a model demonstrating mobility-related impact in PD, which integrated the perspectives of both people with PD and carers. This model illustrates the shared impacts, eg, loss of independence, emotional strain, social isolation and adaption, as well as the unique challenges faced by each group. It also highlights the reciprocal influence between people with PD and their carers.

Clinically, the findings support the need for holistic, person-centred approaches that address not only the physical challenges of mobility loss but also the psychosocial impacts that accompany it. For people with PD, mobility loss is intertwined with identity, social participation and emotional resilience. There is a significant proportion of interventions targeted at people with PD, through national (eg, Parkinsons UK) and international (eg, Michael J Fox Foundation), third sector organisations, and self-management programmes through the digital sphere.^64^ However, carers of people with PD appear not to receive the same attention and opportunities, despite evidence of carer burden, and in contrast to other neurological conditions (eg, dementia) where national care guidelines include specific recommendations for carer wellbeing and support.^65,66^ And yet, we know that carers play a dynamic and imperative role within the care plan for someone with PD.^65^ As this meta-ethnography has shown, mobility loss alone leads to a significant burden for carers coupled with previous reports in the literature of the impact of non-motor and other components of PD and its impacts.^19,25-28,39-41^ These findings show the need for targeted interventions for carers, including education on managing mobility related risks in their care partner, for example falls and falls related consequences and psycho-social support addressing their own health and social isolation.

This review highlights how mobility loss and its fluctuations shape the daily lives of people with PD and carers, influencing independence, safety, social participation and emotional wellbeing. While the included studies did not examine technological solutions directly, the challenges identified, particularly the unpredictability of mobility changes and the impact on planning and safety, suggest potential value in tools that monitor and communicate mobility patterns over time.^67,68^ For example, wearable devices have been explored in other contexts to provide continuous, objective data on movement, supporting more complex assessments.^69^ If designed in collaboration with people with PD and carers, such tools could help better anticipate periods of reduced mobility, support carers in planning assistance and inform more responsive clinical care.^70^ Further qualitative research could explore the acceptability and perceived usefulness of these approaches, ensuring that any future digital innovations are grounded in lived experience.

While most of the included studies focused on the perspectives of people with PD, a smaller number explored carers’ experiences. This imbalance mirrors a broader trend in PD research, where the focus on informal carers is often limited despite their essential role in supporting people with PD.^71^ This pattern is further consistent with wider structural under-recognition of carers within PD care pathways.^65^ Practical and emotional barriers may also contribute to this underrepresentations, as carers frequently report time constraints, stress and priority of caregiving over research involvement.^72^ Together, these challenges likely contribute to the limited qualitative evidence base. Given that mobility loss in PD affects the entire caregiving dynamic, the lack of carer-focused evidence represents a significant gap in the literature and an important area for future research.

### Limitations

The primary limitation of this review is the predominance of studies from Westernised contexts and lack of reporting on inclusion of under-served groups such as ethnic minorities and low socio-economic status, which may not fully capture the diverse cultural and economic dynamics that influence caregiving and the experience of mobility loss in PD. Cultural variations and economic disparities in caregiving roles, health care access, and societal support systems are likely to shape the experience of both people with PD and carers differently.^73,74^ Thus, future research should aim to incorporate a broader range of cultural contexts to gain a more comprehensive understanding of the challenges posed by mobility loss across different populations. Additionally, there is a notable lack of studies involving formal carers, such as professional health care workers. Their perspectives are critical, as they frequently manage mobility impairments in more advanced stages of PD in clinical settings, offering insights into how mobility loss is addressed differently in institutional care environments compared to home settings.^75^ Including formal carers in future research could provide a more complete picture of the caregiving experience and lead to better-informed interventions for both informal and professional carers. We also note that some included studies were published over 20 years ago, and may therefore capture perspectives that do not reflect the current day’s experience of PD. However, including all relevant studies without date restriction allows us to understand the full breadth of literature, and older studies can provide valuable and foundational insights into experiences that remain relevant today.

## Conclusion

This meta-ethnography highlights the significant and multifaceted challenges associated with mobility loss in PD for both people with PD and carers. Mobility impairments not only diminish independence of people with PD but also place a considerable emotional and physical burden on carers. The findings emphasise the interconnected nature of these challenges, revealing that the loss of autonomy and increased dependency due to mobility limitations severely affect the psychosocial wellbeing of both groups. These insights reaffirm the need for capturing real-time data on mobility comprehensive, individualised interventions that address both the physical and emotional aspects of caregiving and patient care in PD. Wearable devices, such as those that monitor DMOs, offer promising avenues to enhance care by providing real-time data on mobility patterns, enabling personalised interventions, and supporting both carers and people with PD in managing mobility fluctuations more effectively. Exploring their potential offers an exciting direction for future research. This review also identifies notable gaps, particularly the lack of representation of non-Western cultural contexts and the absence of perspectives from formal carers. Addressing these gaps in future research is crucial to develop a more globally relevant understanding of caregiving in PD.

## Supplemental Material


Supplemental material - Impact of Mobility Loss on Independence and Psychosocial Wellbeing in People With Parkinson’s Disease and Carers: A Meta-Ethnography
Supplemental material for Impact of Mobility Loss on Independence and Psychosocial Wellbeing in People With Parkinson’s Disease and Carers: A Meta-Ethnography by Megan Hanrahan, Cameron Wilson, Lynn Rochester, Alison Keogh, Ríona Mc Ardle and Katie Brittain in Journal of Geriatric Psychiatry and Neurology

## Data Availability

Data sharing is not applicable to this article as no new data were created or analysed for this study.*
