# Supplementary material for: Impact of Mobility Loss on Independence and Psychosocial Wellbeing in People With Parkinson’s Disease and Carers: A Meta-Ethnography
Source: J Geriatr Psychiatry Neurol. 2026 Jan 7;39(6):683–99. doi: 10.1177/08919887251414664 (PMC13669465; doi:10.1177/08919887251414664)
Supplement: Supplementary file 1 [file sj-pdf-1-jgp-10.1177_08919887251414664.pdf]

NICE Quality Assessment

0-9 (-), 9-18 (+), 19-28 (++)

Author	Theoretical Approach		Study Design	Data Collection	Trustworthiness			Analysis						Ethics	Overall Assessment
	Is a qualitative approach appropriate?	*Is the study clear in what it seeks to do?*	*How defensible/rigorous is the research design/methodology?*	*How well was the data collection carried out?*	*Is the role of the researcher clearly described?*	*Is the context clearly described?*	*Were the methods reliable?*	*Is the data analysis sufficiently rigorous?*	*Is the data 'rich'?*	*Is the analysis reliable?*	*Are the findings convincing?*	*Are the findings relevant to the aims of the study?*	*Conclusions/Is there adequate discussion of any limitations encountered?*	*How clear and coherent is the reporting of ethics?*	*As far as can be ascertained from the paper, how well was the study conducted?*
**Nazzal & Khalil**	++	++	+	++	-	++	++	++	++	++	++	++	++	-	++
**Caap-Ahlgren et al.**	++	++	++	++	-	+	+	++	+	-	++	++	+	++	++
**Davey et al.**	++	++	-	++	-	++	+	+	+	-	++	++	+	+	+
**Hammarlund et al.**	++	+	++	++	-	+	+	++	++	-	++	++	+	+	++
**Johansson et al.**	++	+	++	++	+	+	+	++	++	+	++	++	++	+	++
**Huang et al.**	++	+	+	+	+	++	+	+	++	+	++	++	++	-	++
**LaGrone et al.**	++	+	+	++	-	++	+	++	+	+	++	++	+	-	+

**Owen et al.**	++	+	+	++	-	++	+	+	+	+	++	++	++		+
**Parry et al.**	++	++	++	++	-	++	++	++	++	-	++	++	++	++	++
